# Protective effects of bark ethanolic extract from *Spondias dulcis* Forst F. against DNA damage induced by benzo[*a*]pyrene and cyclophosphamide

**DOI:** 10.1590/1678-4685-GMB-2018-0038

**Published:** 2019-11-14

**Authors:** Caroline de S. Araujo, Lorrane D. Brito, Marina O. Tarifa, Nayara J. Farah da Silva, Karoline S. Rodrigues, Dalita G. S. M. Cavalcante, Andressa S. Gomes, Marcos A. Zocoler, Eidi Yoshihara, Marjori L. Camparoto, Aldo E. Job, Leandra E. Kerche

**Affiliations:** 1 Faculdade de Artes, Ciências, Letras e Educação, Universidade do Oeste Paulista, Presidente Prudente, SP, Brazil.; 2 Faculdade de Medicina, Universidade do Oeste Paulista, Presidente Prudente, SP, Brazil.; 3 Faculdade de Farmácia, Universidade do Oeste Paulista, Presidente Prudente, SP, Brazil.; 4 Departmento de Física, Química e Biologia, Universidade Estadual Paulista “Júlio de Mesquita Filho”, Presidente Prudente, SP, Brazil.; 5 Agência Paulista de Tecnologia dos Agronegócios (APTA), Presidente Prudente, SP, Brazil.

**Keywords:** Comet assay, micronucleus test, Spondias dulcis, cytotoxicity, mutagenicity

## Abstract

This study evaluated the genotoxicity, mutagenicity, antigenotoxicity, and antimutagenicity effects on biochemical parameters of oxidative stress of the *Spondias dulcis* bark ethanolic extract on mice. The extract was evaluated in the doses of 500, 1000, and 1500 mg/kg bw via gavage. To evaluate the protective effects of the extract, benzo[*a*]pyrene (B[*a*]P) and cyclophosphamide (CP) were chosen as DNA damage inducers. Genotoxicity and antigenotoxicity were evaluated by the comet assay. Cytotoxicity, mutagenicity, and antimutagenicity were evaluated by the micronucleus test in bone marrow and peripheral blood. The biochemical parameters of oxidative stress were evaluated by the quantification of catalase activity (CAT) and reduced glutathione (GSH) in total blood, liver and kidney, and malondialdehyde (MDA), in liver and kidney. No genotoxic, cytotoxic, or mutagenic effect was found on mice exposed to the extract. The extract depleted the number of damaged nucleoids in total blood and the number of micronucleus (MN) in both cell types. The extract was able to increase CAT activity and GSH levels and decrease MDA levels after treatment with B[a]P and CP. The results indicate that the *S. dulcis* extract has potential to be used as preventive compound against DNA damage caused by CP and B[a]P.

## Introduction

The use of plants for medical purposes has always been a common practice all over the world. Medicinal plants are considered safe and of low cost, and are known to promote health (Partap *et al.*, 2012). Although some plants possess therapeutic advantages, they can contain harmful, potentially toxic, and mutagenic substances (Lather *et al.*, 2011; Cuyacot *et al.*, 2014; Ferreira-Machado *et al.*, 2014). The Anacardiaceae family is a group of tropical flowering plants that bears drupe fruits. This family comprises several genera that possess economic importance such as the genus *Spondias*. This genus consists of approximately 8 to 12 species that bear edible fruits and is distributed across tropical regions in the world (Narain *et al.*, 2004). *Spondias dulcis* (*S. dulcis*) is a tropical species native from the region that spans from Melanesia through Polynesia, and its fruits are known as cajamanga, Hog plum, or golden apple. *S. dulcis* fruit is commonly used as food, but other parts of this plant are used as remedy. In Cambodia, the bark is used as remedy for diarrhea (Morton, 1987), in eyesight enhancement, and eye infections (Rahmatullah *et al.*, 2009), and the fruit is used for itchiness, internal ulceration, sore throat, and skin inflammation (Wiart, 2006). Certain studies have shown medicinal properties of *S. dulcis*, such as induction of peritoneal macrophages activity (Sarker *et al.*, 2012) and antidiabetic activity (Jantan, 2010). But to date, there is no study evaluating possible cytotoxic and mutagenic effects of *S. dulcis* bark extracts, nor its protective effects against DNA damage induced by chemical compounds.

Different types of mutagenic compounds are present in daily life. Benzo[a]pirene (B[a]P) can be found in many foods, as smoked meat products and cigarette smoke, being related with lung cancer development (Ince *et al.*, 2017; Yao *et al.*, 2017). B[a]P belongs to the group of polycyclic aromatic hydrocarbons (PAHs) and due to its lipophilic nature, B[*a*]P easily crosses the cell membrane and it has to be metabolized in order to be removed from the system. Inside the cell, B[*a*]P is converted into the carcinogen 7,8-dihydrodiol-9,10-epoxide (BPDE) that can form adducts with guanines since it can covalently bind to DNA (IARC Working Group on the Evaluation of Carcinogenic Risks to Humans and International Agency for Research on Cancer, 2010; Shi *et al.*, 2017). Christmann *et al.* (2016) showed that BPDE activates a network of transcriptional alterations of DNA repair and translation DNA synthesis (TLS) genes, leading to protection against cell death and an increased yield of mutations in the survivor cells. As an example, BPDE-DNA adducts in TP53 tumor suppressor gene can induce a mutational profile that is usually found in lung cancer tissue from cigarette smokers (Denissenko *et al.*, 1996). Many of the mutagenic compounds are also used as treatment for various diseases. Cyclophosphamide (CP) is a widely used drug in cancer and non-malign diseases, however it can cause several side effects. (Drimal *et al.*, 2006; Perini *et al.*, 2007; Song *et al.*, 2014). CP affects DNA through its alkylating properties and free radical production (Zhang *et al.*, 2005; Sloczynska et al; 2014). The toxic effects associated with the administration of CP are mainly due to oxidative stress from the increase in the formation of superoxide radicals and hydrogen peroxide (Ettawa *et al.*, 2016; Gunes *et al.*, 2017).

Therefore, the aim of the present study was to evaluate for the first time possible cytotoxic and mutagenic effects as well as the protective effects of *S. dulcis* bark ethanolic extracts *in vivo* on CP- and B[a]P-induced mutagenic damage, using the comet assay and micronucleus test. The extract’ effects on biochemical oxidative stress parameters, such as catalase (CAT), glutathione (GSH), and malondialdehyde (MDA), were assessed in liver, kidney, and total blood.

## Materials and Methods

### Chemicals

Cyclophosphamide (CP) (CAS: 50-18-0) and benzo[*a*]pirene (B[*a*]P) (CAS: 50-32-8) were purchased from Sigma Chemical Co. (St. Louis, MO), diluted in distilled water and saline, respectively, and used as positive controls and as DNA damage-inducing agents in the tests concerning possible protective effects.

### Plant material and extract preparation

A voucher of the plant is deposited in the Universidade Estadual de Maringá herbarium with the code HUEM 24319. The fresh stem bark of *S. dulcis* were washed with water immediately after collection in the plant nursery of Universidade do Oeste Paulista, Presidente Prudente, Brazil (UNOESTE). Then, the bark was chopped into small pieces, air dried at room temperature for about 10 days, and turned into powder (1 kg), which was infused in 6 L of pure ethanol for 7 days at room temperature (23 ºC ± 5). After 7 days, the extract was filtered through cotton plugs and then through a Whatman No. 1 filter paper. The extract was concentrated under reduced pressure below 50 ºC through rotatory vacuum evaporator (RE200 Sterling, UK). The concentrated extract was stored at 4 ºC.

### Pharmacognostic characterization

After extraction, thin layer chromatography (TLC) was carried out on silica gel plates with fluorescence indicator (Macherey-Nagel), according to Wagner and Bladt (2001). The solvent system was ethyl acetate, acetic acid, formic acid, distilled water (100:11:11:26) eluted with filter paper. After the elution of the extracts, the plates were dried out in room temperature, revealed with diphenylboryloxyethylamine (NP) (Sigma-Aldrich), and placed under an UV lamp (365 nm).

The total phenolic content of the extract was determined by the Folin-Ciocalteu method (Kaur and Kapoor, 2002). Briefly, 200 μL of crude extract (1 mg/mL) was added to 3 mL of distilled water, mixed thoroughly with 0.5 mL of Folin-Ciocalteu reagent for 3 min, followed by the addition of 2 mL of 20% (w/v) sodium carbonate. The mixture was allowed to stand for 60 min in the dark, and absorbance was measured at 650 nm. The total phenolic content was calculated from the calibration curve, and the results were reported as mg of gallic acid equivalent per g dry weight.

The total flavonoid content of crude extract was determined according to Rolim *et al.* (2005) with modifications. The concentration of total flavonoids was determined spectrophotometrically as rutin equivalents compared to the standard curve of rutin. Standard rutin (10 mg) was dissolved in methanol and acetic acid 0.02 M (99:1) to concentrations of 12.5, 25, 50, 75 and 85 mg/mL. The absorbance measurements were obtained at 361 nm. The methanol and acetic acid mixture was used as a solvent for the preparation of the sample solution. Analysis was performed in triplicates and the results are presented as % of total flavonoids.

### Animals

The experimental protocols for this study were approved by the Local Ethics Committee for Animal Use (CEUA) of UNOESTE, register No. 2425/2015. Male and female albino Swiss mice (*Mus musculus*), aged 7-8 weeks and weighing ~30 g at the beginning of the experiments, were used for the comet assay, micronucleus test, and biochemical parameters analysis. They were obtained from the mouse-breeding colony at the Universidade do Oeste Paulista and were kept individually in polypropylene cages following the conditions for animal care recommended by the Canadian Council on Animal Care (Olfert *et al.*, 1993). The mice (*n* = 120) were divided into groups of 10 (five males and five females) for each treatment and these mice were the same for all the end points (comet assay, micronucleus test, and biochemical parameters).

### Experimental design

The mice received water and food *ad libitum* throughout the treatment period (24 h). Firstly, the biological effects (cytotoxic, genotoxic, mutagenic, and oxidative stress generation) of *S. dulcis* extracts were evaluated at three acute doses: 500, 1000, and 1500 mg/kg, once via gavage. Since no other study evaluate these parameters, these doses were based on the solubility of the bark extract in distilled water. A negative control group with distilled water and two positive control groups with CP (40 mg/kg bw) and B[a]P (9 mg/kg bw) were established.

The protective effects of the extract was also assessed. For this, CP (40 mg/kg bw) was administrated in a single i.p. dose 1 h after the plant extract was administered via gavage, and B[*a*]P (9 mg/kg bw) was administrated in a single s.c. dose simultaneously to the administration of the extract via gavage. A negative control group was established with distilled water.

After the treatment period (24 h), the mice were put under anesthesia with 10% chloral hydrate i.p. (4 mL/kg bw), total blood was collected from the heart and peripheral blood cells were collected from the tail vein. The animals were euthanized by cervical dislocation, and liver, kidneys, and femurs were collected.

### Cytotoxicity and micronucleus test in mouse bone marrow

Bone marrow was removed 24 h after the treatment. Briefly, the bone marrow was flushed out of the femurs in a centrifuge tube with fetal calf serum. The bone marrow cells were collected by centrifugation at 1000 rpm for 10 min, and the pellet was resuspended in 0.3 mL of supernatant for the slide preparation. A drop of the suspension was smeared on a clean slide, air-dried, fixed in absolute methanol for 10 min, and stained in the following day with Giemsa (diluted with phosphate buffer, pH 6.8).

Cytotoxicity was measured using the percentage of polychromatic erythrocytes (PCEs) among 1000 erythrocytes (PCEs/PCEs + normochromatic erythrocytes (NCEs)). For the micronucleus test, three thousand PCEs were analyzed (1000 per slide in triplicate), and the number of micronucleated PCEs (MNPCEs) was recorded (Schmid, 1975). The percentage of reduction in micronucleated cells (%R) was calculated using the formula: %R = [(mean in A – mean in B) / (mean in A – mean in C)] 100, where A is the group treated with positive control (CP or B[*a*]P), B is the group treated with different doses of the extract plus positive control, and C represents the group treated with distilled water (negative control group) (Waters *et al.*, 1990).

### Comet assay and micronucleus test of peripheral blood cells

The comet assay (pH > 13) was conducted according to the protocols of Tice *et al.* (2000) and Singh *et al.* (1988). The comet assay detects initial and/or acute DNA damage even after short exposures. Peripheral blood (10 μL) was mixed with low melting point agarose (0.5%), 180 μL, and spread onto microscope slides precoated with normal melting point agarose (1.5%) constituting a slide with two layers of agarose. The cells were covered with a coverslip and maintained at 4 ºC for 10 min. Coverslips were removed ant the slides were immersed in a freshly prepared lysis solution consisting of 2.5 M NaCl, 100 mM ethylenediaminetetraacetic acid (EDTA), 10% dimethylsulfoxide, 1% Triton X-100, and 10 mM Tris, pH 10, for 60 min at 4 ºC. After lysis, the slides were placed in a horizontal electrophoresis unit containing 300 mM NaOH and 1 mM EDTA at pH > 13 and left for 20 min to denature the DNA. Electrophoresis was run for 20 min at 1 V/cm (25 V and 300 mA). Slides were subsequently immersed in a neutralization buffer (0.4 M Tris – HCl, pH 7.5) for 15 min. After being dried at ambient temperature, slides were fixed in ethanol for 5 min and stored until analysis. After removal from storage, each slide was stained with 30 μL 4’,6-Diamine-2’-phenylindole dihydrochloride (DAPI) (1 mg/mL) and immediately analyzed in a fluorescence microscopy (Olympus). For each treatment, the extent and distribution of DNA damage indicated by the comet assay was evaluated by examining 100 randomly-selected and non-overlapping cells on the slides (i.e., 300 cells per treatment). On each slide, the cells were visually scored and allocated to one of four classes (0, 1, 2, and 3) (Speit and Hartmann, 2005) and the total score for 300 comets was obtained according to the formula of Manoharan and Banerjee (1985), as shown below:

$$Score=\left(ln_{1}+2n_{2}+3n_{3}\right)$$

where *n* is the number of cells in each class analyzed. The total score could therefore range from 0 to 300.

The micronucleus test on peripheral blood cells was performed according to the protocol described by Hayashi *et al.* (1990), which uses slides prestained with acridine orange. Blood sampling was performed 24 h after the treatment. The sample was placed in the center of a prestained slide and covered with a coverslip (24 50 mm). The slides were stored in the dark at -20 ºC until the cytological examination was performed. The cell preparations were examined under a fluorescence microscope (Olympus) with a blue (488 nm) excitation filter and a yellow (515 nm) emission (barrier) filter using an immersion objective. A total of 3000 reticulocytes per treated animal were analyzed (1000 per slide in triplicate), and the number of micronucleated reticulocytes (MNRETs) was counted. The percentage of reduction in micronucleated cells (%R) was calculated using the formula: %R = [(mean in A – mean in B) / (mean in A – mean in C)] 100, where A is the group treated with positive control (CP or B[*a*]P), B is the group treated with different doses of the extract plus positive control, and C represents the group treated with distilled water (negative control group) (Waters *et al.*, 1990).

### Evaluation of CAT activity and GSH concentrations in total blood

For CAT analysis assay, the hemolysate was diluted 1:20 in 0.1 M phosphate buffer, pH 7.0, and the assay was performed in triplicate. CAT activity was measured in 50 mM phosphate buffer (pH 7.0) by monitoring the decrease in absorbance at 240 nm for 30 s after the addition of 10 mM hydrogen peroxide. One unit of CAT activity is the amount of enzyme that decomposes 1 μM H_2_O_2_ per min at 25 ºC (Aebi, 1984). The erythrocyte CAT activity was expressed as κ/g protein.min^-1^.

Reduced GSH concentrations were estimated using the method of Ellman (1959). Briefly, erythrocytes (0.3 mL) were hemolyzed using 10% Triton X-100 (0.1 mL) and precipitated with 200 μL of 20% trichloroacetic acid (TCA). After centrifugation at 5,000 rpm for 10 min, color was developed in the supernatant by adding 50 μL of 10 mM 5-5’-dithio-bis (2-nitrobenzoic acid) (DTNB), and the optical density was recorded at 412 nm using α-cysteine as reference standard. The GSH content was expressed as nanomoles per milliliter (nM/mL) of blood.

### TBARS and GSH levels in kidney and liver

Thiobarbituric acid reactive substances (TBARS) measurements were done according to the method of Uchiyama and Mihara (1978) with modifications. Briefly, kidney or liver tissue (200 mg) was homogenized in 5.0 mL ice-cold 1.15% KCl, and an aliquot (200 μL) of homogenate was mixed with 400 μL of the TBA solution (1% TBA, 50 mM NaOH and 0.1 mM BHT) and 200 μL of 7% phosphoric acid. The mixtures were incubated in a boiling bath for 15 min. After cooling the tubes on ice, 1.5 mL of *n*-butanol was added and the reaction mixture was centrifuged at 6,000 rpm for 10 min. The absorbance of the supernatant was read at 532 nm, and the TBARS concentrations were calculated using tetraethoxy propane as a reference standard. The TBARS concentrations in tissue was reported as nmol MDA/g of tissue.

Reduced GSH concentrations were estimated using the method of Ellman (1959). Briefly, the same homogenized kidney or liver tissue cited above was diluted in water (1:4), precipitated with 50% TCA and centrifuged at 6,000 rpm for 10 min. An aliquot (500 μL) of the supernatant was added to 2.0 ml Tris-EDTA buffer (0.2 M, pH 8.9) and 100 μL of 0.01 M DTNB in methanol. The solution was incubated at room temperature for 15 min and read at 412 nm using α-cysteine as reference standard (Sedlak and Lindsay, 1968). The results were reported as nmol GSH/g of tissue.

### Statistical analysis

All results are reported as mean ± standard deviation (SD). No differences were observed between males and females for all parameters analyzed, and thus, means were calculated for all treated groups using both females and males (Student’s *t*-test, *p*>0.05). The results of the cytotoxicity were compared between the treatment groups and the negative control group using one-way ANOVA and Dunnett’s test (at significance level *p*<0.05) in GraphPad Prism 6 (GraphPad Software, USA). For assessment of the biochemical parameters, mutagenicity, and protective effects, all groups were compared using one-way ANOVA and Tukey’s test (*p* < 0.05).

## Results

### Pharmacognostic characterization

**Figure 1:**
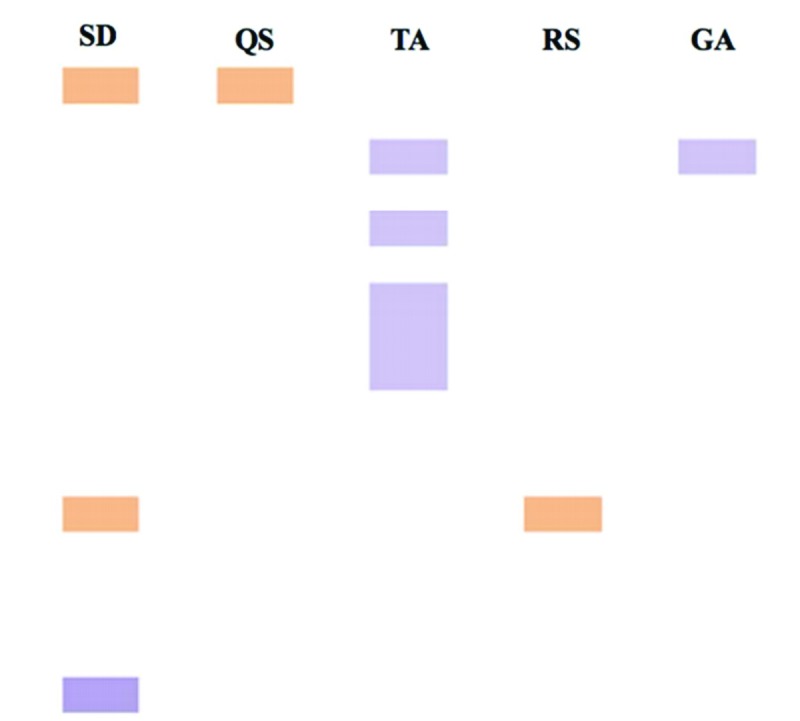
Thin layer chromatography of *Spondias dulcis* Forst F bark ethanolic extract. SD, *S. dulcis* extract; QS, Quercetin standard; TA, Tannic acid standard; RS, Rutin standard; GA, Gallic acid standard.

The TLC of the extract was carried out using flavonoids (quercetin and rutin) and tannins (tannic and gallic acid) as references, and the results showed that this extract presents quercetin and rutin in its constitution (Figure 1).

The analysis of total phenolic content of the extract showed a concentration of 518 mg of phenolic acids in 1 g of the extract, based on gallic acid standard curve (y = 0.007x + 0.0027, R^2^ = 0.9978). The analysis of total flavonoid content of the extract showed a concentration of 185 mg of flavonoids in 1 g of the extract, based on rutin standard curve (y = 0.0154x – 0.0044, R^2^ = 0.9926).

### Mouse bone marrow

#### Cytotoxicity

**Figure 2:**
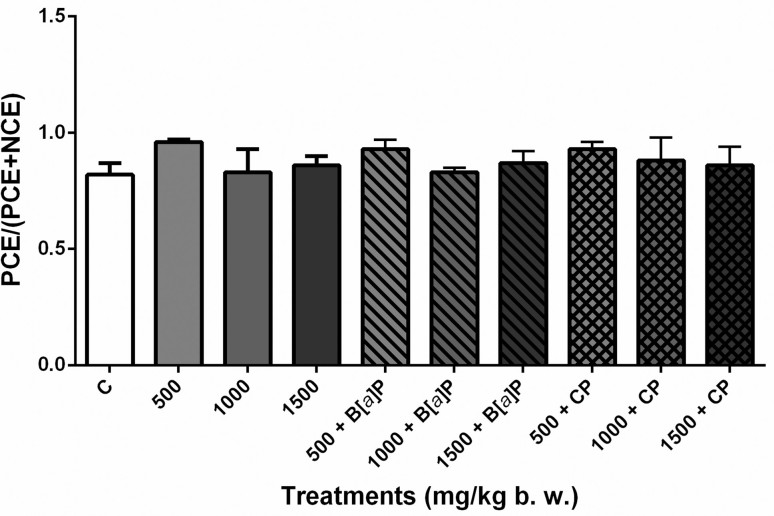
Evaluation of cytotoxicity in mouse bone marrow cells after acute treatment with *S. dulcis* bark ethanolic extract (500, 1000, and 1500 mg/kg bw) associated or not with B[*a*]P and CP. Calculation of PCE/(PCE+NCE) on examination of 1000 erythrocytes. Shown are means ± SD for 10 animals (male and female) from each treatment. ANOVA and Dunnett’s test (*p*<0.05). PCE: polychromatic erythrocyte; NCE: normochromatic erythrocyte; C: distilled water; B[*a*]P: benzo[*a*]pyrene; CP: cyclophosphamide.

Results from the ANOVA and Dunnett’s test showed no significative difference between all treated groups and the negative control group (distilled water). Therefore, these concentrations of the extract (500, 1000, and 1500 mg/kg bw), associated or not to the positive controls (CP and B[*a*]P), had no cytotoxic effects in the bone marrow of mice (*p*<0.05) (Figure 2).

#### Micronucleus

**Figure 3:**
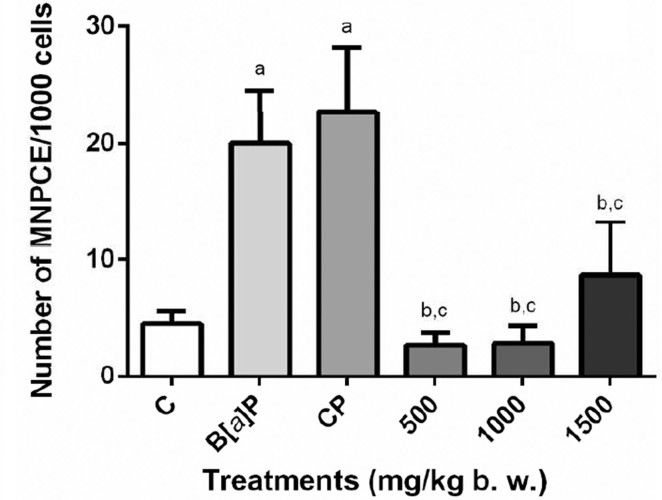
Frequency of MNPCEs in mouse bone marrow after acute treatment with three different concentrations (500, 1000, and 1500 mg/kg bw) of the *S. dulcis* bark ethanolic extract. Shown are the means ± SD for 10 animals (male and female) from each treatment. Statistical analysis was performed using ANOVA and Tukey’s test (*p*<0.05). ^a^Statistically different from negative control group; ^b^ statistically different from B[*a*]P control group. ^c^Statistically different from CP control group. MNPCE: micronucleated polychromatic erythrocyte; C: distilled water; B[*a*]P: benzo[*a*]pyrene; CP: cyclophosphamide.

In bone marrow, the number of micronuclei did not change after treatment with either 500, 1000, and 1500 mg/kg bw of the extract (Figure 3). The bone marrow was also used to analyze protective effects of *S. dulcis* ethanolic bark extract. A large protective effect can be observed for both DNA damage inducers (B[*a*]P and CP). In bone marrow, the greatest protection occurred for the dose 500 mg/kg bw associated with CP (87.18%) (Table 1).

**Table 1:** Numbers of micronucleated PCEs (MNPCE) from mouse bone marrow and percent damage reduction (%R) obtained in the determination of *in vivo* antimutagenicity of *Spondias dulcis* Forst F. bark ethanolic extract at three concentrations, and the respective controls.

Treatments (mg/kg b.w.)	No. animals	MNPCE (*X* ± SD)	% R
C	10	4.50 ± 1.05^a^	-
B[*a*]P	10	20.00 ± 4.45^b^	-
CP	10	22.67 ± 5.50^c^	-
500 + B[*a*]P	10	8.33 ± 1.51^a^	75.29%
1000 + B[*a*]P	10	9.17 ± 3.25^a^	69.87%
1500 + B[*a*]P	10	9.17 ± 1.52^a^	69.87%
500 + CP	10	6.83 ± 0.79^a^	87.18%
1000 + CP	10	8.00 ± 1.90^a^	80.74%
1500 + CP	10	8.33 ± 1.96^a^	78.92%

### Peripheral blood

#### Comet assay

Results on DNA stability are shown in Table 2. Exposure to B[*a*]P and CP increased the number of damaged nucleoids while no evidence of genotoxic effect was seen after the exposure of the cells to *S. dulcis* extract (500, 1000 and 1500 μg/mL) under the conditions of the test. The results of experiments in which the protective properties of the *S. dulcis* extract on DNA damage induced by B[a]P and CP are summarized in Table 3. All doses tested were able to reduce the number of damaged nucleoids to levels similar to the negative control

**Table 2:** Number of nucleoids observed in each comet class in 300 cells analyzed per treatment, and their respective mean scores when assessing the genotoxicity of *Spondias dulcis* at three different concentrations in mice.

Treatment (μg/mL)	Comet Class				Damaged nucleoids	Score	*X* ± SD
	0	1	2	3			
Control	99	1	0	0	1	1	1.33 ± 0.58
	98	2	0	0	2	2	
	99	1	0	0	1	1	
CP	66	28	3	3	34	43	44.0 ± 6.56^a^
	66	30	4	0	34	38	
	55	39	6	0	45	51	
B[*a*]P	87	13	0	0	13	13	12.67 ± 1.53^a^
	87	12	1	0	13	14	
	90	9	1	0	10	11	
*Spondias dulcis*							
500	98	2	0	0	2	2	2.330.58^b c^
	97	3	0	0	3	3	
	98	2	0	0	2	2	
1000	98	2	0	0	2	2	2.33 ± 1.53^b c^
	97	2	1	0	2	4	
	99	1	0	0	1	1	
1500	99	1	0	0	1	1	2.33 ± 1.53^b c^
	97	2	1	0	3	4	
	98	2	0	0	2	2	

**Table 3:** Number of nucleoids observed in each comet class in 300 cells analyzed per treatment, and their respective mean scores when assessing the antigenotoxicity effect of *Spondias dulcis* at three different concentrations in mice.

Treatment (μg/mL)	Comet Class				Damaged nucleoids	Score	*X* ± SD
	0	1	2	3			
Control	99	1	0	0	1	1	1.33 ± 0.58
	98	2	0	0	2	2	
	99	1	0	0	1	1	
CP	66	28	3	3	34	43	44.0 ± 6.56^a^
	66	30	4	0	34	38	
	55	39	6	0	45	51	
B[*a*]P	87	13	0	0	13	13	12.67 ± 1.53^a^
	87	12	1	0	13	14	
	90	9	1	0	10	11	
*Spondias dulcis* + CP							
500	96	4	0	0	4	4	7.0 ± 3.0^a b^
	96	3	1	0	4	7	
	90	10	0	0	10	10	
1000	99	1	0	0	1	1	3.0 ± 2.0^b^
	98	1	1	0	2	3	
	95	5	0	0	5	5	
1500	95	4	1	0	5	6	5.670.58^b^
	94	6	0	0	6	6	
	95	5	0	0	5	5	
*Spondias dulcis* + B[*a*]P							
	96	4	0	0	4	4	
500	100	0	0	0	0	0	2.3 ± 2.1^c^
	97	3	0	0	3	3	
	92	5	3	0	8	11	
1000	97	2	1	0	3	4	7.0 ± 3.6^a c^
	94	6	0	0	1	6	
	97	2	1	0	3	4	
1500	95	5	0	0	0	5	6.7 ± 3.8^a c^
	90	9	1	0	2	11	

#### Micronucleus

**Figure 4:**
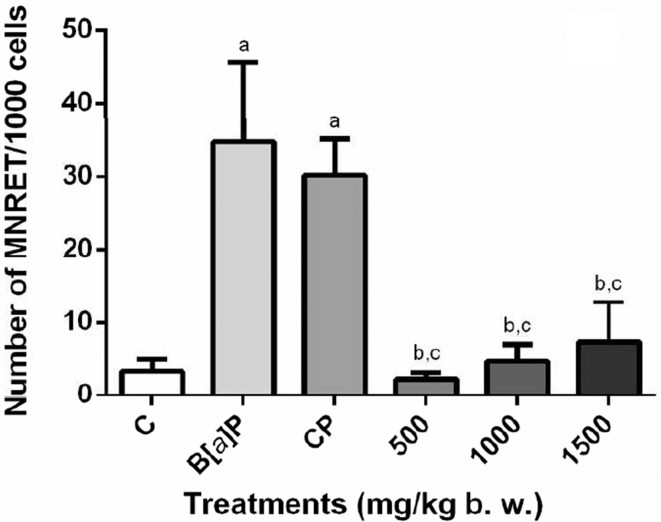
Frequency of MNRETs in mouse peripheral blood after acute treatment with three different concentrations (500, 1000, and 1500 mg/kg bw) of the *S. dulcis* bark ethanolic extract. The plot shown the means ± SD for ten animals (male and female) from each treatment. Statistical analysis was performed using ANOVA and Tukey’s test (*p*<0.05). ^a^ Statistically different from negative control group. ^b^ Statistically different from B[*a*]P control group; ^c^ statistically different from CP control group. MNRET: micronucleated reticulocyte; C: distilled water; B[*a*]P: benzo[*a*]pyrene; CP: cyclophosphamide.

The number of micronuclei in blood induced by the extract from *S. dulcis* is in the same range as distilled water, demonstrating that the extract has no genotoxic effects in acute treatment. On the other hand, administration of B[*a*]P and CP resulted in a significant increase of micronucleus. Peripheral blood was also used to analyze protective effects of *S. dulcis* (Figure 4). A great protective effect can be observed for both DNA damage inducers (B[*a*]P and CP). In peripheral blood, the greatest protection occurred for the dose of 500 mg/kg bw associated with B[a]P (98%) (*p* < 0.05) (Table 4).

**Table 4:** Numbers of micronucleated RETs (MNRET) from peripheral blood and percent damage reduction (%R) obtained in the determination of *in vivo* antimutagenicity of *Spondias dulcis* Forst F. bark ethanolic extract at three concentrations, and the respective controls.

Treatments (mg/kg b.w.)	N. of animals	MNRET (*X* ± SD)	% R
C	10	3.33 ± 1.63^a^	-
B[a]P	10	30.20 ± 10.87^b^	-
CP	10	34.75 ± 4.97^c^	-
500 + B[*a*]P	10	3.75 ± 0.96^a^	98.00%
1000 + B[*a*]P	10	5.00 ± 1.47^a^	93.78%
1500 + B[*a*]P	10	5.33 ± 1.03^a^	92.56%
500 + CP	10	7.80 ± 1.63^a^	85.77%
1000 + CP	10	8.17 ± 2.40^a^	84.60%
1500 + CP	10	10.33 ± 2.39^d^	77.72%

### CAT and GSH in total blood

**Figure 5:**
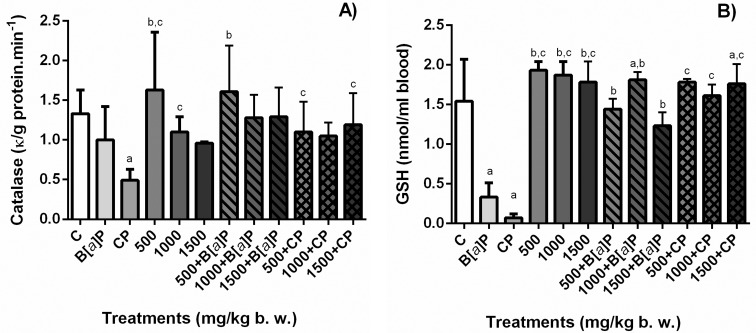
Quantification of CAT (A) and GSH (B) in total blood of mouse treated with *S. dulcis* bark ethanolic extract (500, 1000, and 1500 mg/kg bw) associated or not to the positive controls (B[*a*]P and CP). Results are shown as means ± SD. ^a^ Statistically different from negative control group; ^b^ statistically different from B[a]P control group; ^c^ statistically different from CP control group. CAT: catalase; GSH: glutathione; C: distilled water; B[*a*]P: benzo[*a*]pyrene; CP: cyclophosphamide. Statistical analysis performed using ANOVA and Tukey’s test with significance threshold of *p*<0.05.

Figure 5 shows CAT activity and GSH concentration among the different groups. None of the three doses of the extract (500, 1000, and 1500 mg/kg bw) altered CAT activity and GSH levels in erythrocytes. B[a]P and CP treatments depleted severely the CAT activity and quantity of GSH in the erythrocytes, and the association of the extract in the doses of 500 and 1000 mg/kg bw with these chemicals (B[*a*]P and CP) increased the activity of CAT and the levels of GSH in the erythrocytes similarly to the negative control (*p* < 0.05). 

### TBARS and GSH in tissue

**Figure 6:**
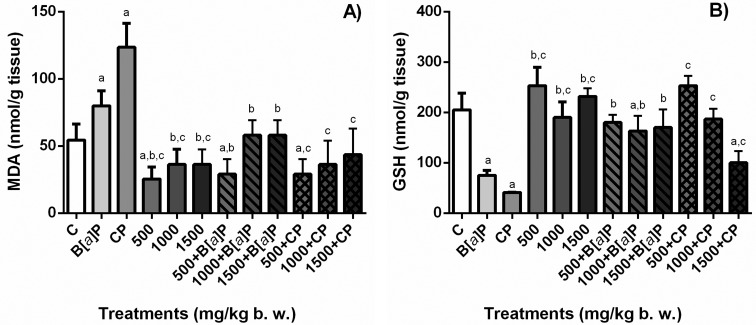
Quantification of lipoperoxidation by measuring the formation of MDA, principal thiobarbituric acid-reactive specie (TBARS) (A) and GSH (B) in the liver of mouse treated with *S. dulcis* bark ethanolic extract (500, 1000, and 1500 mg/kg bw) associated or not to the positive controls (B[*a*]P and CP). Results are shown as means ± SD. ^a^ Statistically different from negative control group; ^b^ statistically different from B[a]P control group; ^c^ statistically different from CP control group. MDA: malondialdehyde; GSH: glutathione; C: distilled water; B[*a*]P: benzo[*a*]pyrene; CP: cyclophosphamide. Statistical analysis performed using ANOVA and Tukey’s test with significance threshold of *p*<0.05.

**Figure 7:**
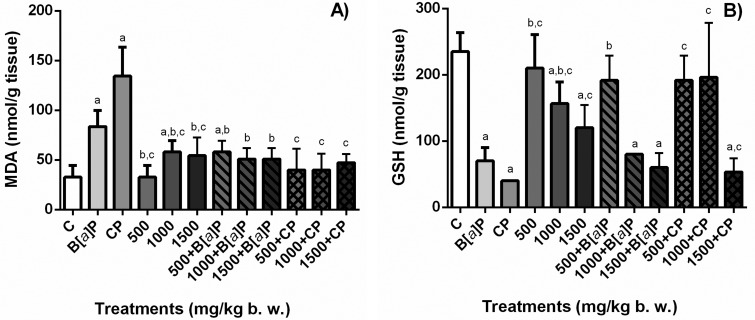
Quantification of lipoperoxidation by measuring the formation of MDA, principal thiobarbituric acid-reactive specie (TBARS) (A) and GSH (B) in the kidney of mouse treated with *S. dulcis* bark ethanolic extract (500, 1000, and 1500 mg/kg bw) associated or not to the positive controls (B[*a*]P and CP). Results are shown as means ± SD. ^a^ Statistically different from negative control group; ^b^ statistically different from B[a]P control group; ^c^ statistically different from CPA control group. MDA: malondialdehyde; GSH: glutathione; C: distilled water; B[*a*]P: benzo[*a*]pyrene; CP: cyclophosphamide. Statistical analysis performed using ANOVA and Tukey’s test with significance threshold of *p*<0.05.

The levels of the oxidative stress biomarkers TBARS and GSH in liver and TBARS and GSH in kidney are shown in Figures 6 and 7, respectively.

In the liver, mice treated with the concentrations of 500 and 1500 mg/kg bw of the extract did not show an increase in lipid peroxidation that was measured by the amount of MDA in the tissue (*p*<0.05). On the other hand, B[*a*]P and CP treatments enhanced the levels of MDA in the liver, and the association of the extract to these chemicals depleted the levels of MDA in the tissue to negative control levels (*p*<0.05). In the kidney, the levels of MDA for the animals treated with the extract associated or not to the positive controls were similar to the negative control (*p*<0.05). These results show that the *S. dulcis* extract significantly prevented the peroxidative effects of B[*a*]P and CP.

The treatment with B[*a*]P and CP severely depleted GSH levels in liver tissue. For the treatment with the extract the only dose that did not deplete the levels of GSH, associated or not the positive controls B[*a*]P and CP, and maintained the levels similar to the negative control, was 500 mg/kg bw. While all the concentrations tested did not deplete the blood GSH levels, the results were different for the liver. In the kidney, all three concentrations tested (500, 1000, and 1500 mg/kg bw) maintained the levels of GSH in the tissue, and B[*a*]P and CP associated to the extract from *S. dulcis* was able to enhance the levels of GSH.

## Discussion

It is very well known that phenolic compounds have the potential to exhibit multiple biological effects, including antioxidant activity (Huda-Faujan *et al.*, 2009; Huang *et al.*, 2016). According to various studies, phenolic compounds are effective in preventing many pathologies, such as cancer (Mocanu *et al.*, 2015), inflammation (Biasi *et al.*, 2011), diabetes (Szkudelski and Szkudelska, 2015), and cardiovascular diseases (Yamagata *et al.*, 2015). The pharmacognostic characterization of the *S. dulcis* bark ethanolic extract showed the presence of flavonoids and phenolic acids in its constitution. Because of their chemical characteristics, phenolic acids can usually elicit primary prevention inhibiting mutations and cancer initiation in extracellular and intracellular media, preventing the uptake of mutagens and carcinogens, maintaining the DNA structure safe (De Flora and Ferguson, 2005). Flavonoids are composed of three rings (A, B, and C) and the substitution of hydroxyl groups in these rings results in the successful scavenge of ROS, increasing their antioxidant capacity (Amic *et al.*, 2007). Quercetin, one of the flavonoids found in the extract, is very active due to free 3-OH groups that stabilize the B-ring for free radical scavenging ability (Rice-Evans *et al.*, 1996). Other studies have proven the ability of quercetin to reduce DNA damage provoked by B[*a*]P and CP (Hollman *et al.*, 1997; Wilms *et al.*, 2005; Abo-Zeid *et al.*, 2018).

Anomalies and initial lesions on DNA, such as single and double strand breaks and crosslinks, can be detected by the comet assay. These DNA damages can either be repaired or lead to cytotoxicity and mutations, and the comet assay detects these lesions in early stages. B[*a*]P and CP were chosen as positive controls, as they can elicit DNA damage. As expected, B[*a*]P and CP were genotoxic to the mouse cells, as indicated by the rise in the number of damaged nucleoids found in the comet assay. On the other hand, the extract of *S. dulcis* did not raise the number of damaged nucleoids, and it was able to prevent the DNA damage caused by B[*a*]P and CP. The extract of *S. dulcis* probably prevented DNA damage in the mouse cells due to the presence of flavonoids and phenolic acids in its composition. Other works have shown the effects of these polyphenols in the prevention of lesions on DNA that could result in carcinogenesis (Guo *et al.*, 1996; Amararathna *et al.*, 2016). The hydroxyl groups present in these polyphenols have the ability to donate hydrogen atoms, converting free radicals into chemically stable molecules, preventing DNA damage. These groups are also able to bond with biological membranes altering receptors and enzymes. Quercetin, a flavonoid found in the extract, has been pointed as a potent antagonist for the AhR and for the transcription factor involved in the activation of CYP enzymes (Denison *et al.*, 2002, 2003; Murakami *et al.*, 2008).

Permanent damage caused by adducts and strand breaks in DNA can be evaluated by the use of the micronucleus test. In bone marrow and in peripheral blood, the frequency of micronucleated cells was evaluated 24 h after the treatment. This time point was chosen based on the kinetics of micronucleus formation and cell migration (Vikram *et al.*, 2007). From our work it is possible to conclude that the *S. dulcis* extract was not mutagenic, since after the treatment the experimental group showed the same number of micronucleated cells as the negative control group for both cell types. By using the micronucleus test it was also possible to assess the toxicity of the extract from the ratio PCE/(PCE+NCE), since the appearance of polychromatic (PCE) or normochromatic erythrocytes (NCE) in bone marrow is an important indicator of cytotoxicity (Venkatesh *et al.*, 2007). This ratio is important because it indicates the acceleration or inhibition of erythropoiesis, and a decline in this ratio suggests cytotoxicity of the test compound (Al-Harbi, 1993). Our results showed that none of the doses of the extract tested were cytotoxic, including in association with B[*a*]P and CP. In the antimutagenicity evaluation, as expected, B[*a*]P and CP increased the number of micronuclei in bone marrow and peripheral blood after their administration, when compared to the negative control. However, the results show that the extract associated with B[*a*]P and CP caused a significant reduction in the mean MNPCE and MNRET in the protocol used in this study.

Polyphenols also explain the enhancement of CAT activity in total blood and GSH levels in total blood, liver, and kidney after the treatment that associated B[*a*]P and CP with the different doses of the extract. CAT is an enzyme ubiquitously found in cells, with the function to decompose hydrogen peroxide into water and oxygen (Majumder *et al.*, 2017). The treatment with B[*a*]P and CP reduced CAT activity, probably due to the release of hydrogen peroxide that led to the consumption of the enzyme. However, when the animals were treated with the extract of *S. dulcis* associated to the positive controls, CAT activity was restored to similar levels as those in the negative control. GSH (γ-L-glutamyl-L-cysteinylglycine) is one of the most important redox agents of aerobic organisms, as it serves as a ubiquitous nucleophile that converts a variety of electrophilic substances under physiological conditions. GSH mainly serves as a reducing agent for hydroperoxides (Deponte, 2013). The depletion of GSH levels after the treatment with B[*a*]P and CP, probably occurred because of the production of ROS, specially hydrogen peroxide, that led to GSH consumption. The ability of flavonoids to quench hydrogen peroxide was shown in previous studies (Bors *et al.*, 1990; Pignatelli *et al.*, 2000). Since flavonoids and phenolic acids are present in the *S. dulcis* extract, and they have the ability to quench hydrogen peroxide elicited by B[*a*]P and CP metabolites, they probably acted synergistically, depleting the consumption of CAT in total blood and GSH in the erythrocytes, liver, and kidney cells.

TBARS have been employed to detect and quantify lipid peroxidation in a variety of chemical as well as biological matrices (Janero, 1990). The treatment with B[*a*]P and CP enhanced the levels of MDA in liver and kidney, respectively, and the formation of ROS from B[*a*]P and CP metabolites, specially hydrogen peroxide, probably enhanced lipid peroxidation in these organs. It is worthy of note that the treatment with *S. dulcis* extract did not raise significantly the levels of MDA in liver and kidney. The association of the extract with B[*a*]P and CP depleted the levels of MDA significantly. Once again, polyphenols in the extract composition are probably the responsible agent for the depletion in MDA levels in liver and kidney. Other studies have shown that polyphenols can directly lower MDA levels in different organs (Dogan *et al.*, 2013; Miao *et al.*, 2014; Sen *et al.*, 2015) and quercetin is able to suppress lipoperoxidation in the liver (Chowdhury and Giri, 2017).

From the results on protective effects of the *S. dulcis* extract it can be seen that the dose with the greatest effects was the lowest one 500 mg/kg bw). This is called an inverse dose-response, and it is very complex to analyze, since in a plant extracts several chemopreventive and antioxidant compounds can act synergistically (Knasmüller *et al.*, 2002). Some of the phenolic compounds found in plant extracts can act as pro-oxidant under specific conditions (Dai and Mumper, 2010). *In vivo* conditions can also present divergent responses in different organs and cells, and other studies have also shown protective effects in an inverse dose-response manner, both in *in vivo* and *in vitro* conditions (Antunes and Takahashi, 1998; Ananthi *et al.*, 2010; Tavares *et al.*, 2011; Alves *et al.*, 2013).

De Flora and Ferguson (2005) suggested that the antimutagenicity effects related to plant extracts could be due to: (i) inhibition of the DNA damage inducer uptake; (ii) binding to the DNA damage inducer inside or outside the cells, preventing its action on DNA; (iii) increasing levels of endogen antioxidant; and (iv) increasing the maintenance of DNA structure and modulation of DNA metabolism and repair. Based on the results found in this study, where polyphenols were found in the *S. dulcis* extract and both B[*a*]P and CP induced DNA damage mainly by the induction of ROS production, we propose that *S. dulcis* bark ethanolic extract acted by increasing the levels of endogen antioxidant(s) in the mouse cells, contributing with the antimutagenic effect seen for *S. dulcis* extract.

In conclusion, we have shown for the first time that the *Spondias dulcis* bark ethanolic extract does not show cytotoxic, genotoxic, or mutagenic activity in bone marrow and peripheral blood of mice. This extract presented protective effects against B[*a*]P- and CP-induced DNA damage, depleting the number of damaged nucleoids, MNPCE, and MNRET in these animals. This extract also increased CAT activity in blood and GSH levels in blood, liver, and kidney, and depleted MDA levels in liver and kidney of animals treated with B[*a*]P and CP. The results obtained in this study indicate that the *Spondias dulcis* extract could be useful as preventive compound against DNA damage caused by mutagenic agents CP and B[*a*]P. More studies are being conducted in order to clarify and isolate the compounds of the *Spondias dulcis* extract and to elucidate their properties.
